# The Role of Tumor Deposits in Predicting the Efficacy of Chemotherapy in Stage III Colon Cancer

**DOI:** 10.3389/fonc.2020.586603

**Published:** 2020-10-14

**Authors:** Mingyu Shi, Hongzhi Zhang, Guozhong Yao, Jianjun Wu, Chuming Zhu, Xu Zhang, Yuan Ren

**Affiliations:** Department of General Surgery, Liyang People’s Hospital, Liyang Branch of Jiangsu Provincial People’s Hospital, Liyang, China

**Keywords:** tumor deposits, stage III, colon cancer, chemotherapy, survival

## Abstract

**Purpose:**

To evaluate the role of tumor deposits (TDs) in predicting the efficacy of chemotherapy in stage III colon cancer.

**Methods:**

Using the SEER^∗^Stat software Version 8.3.6, we started with a national cohort of colon cancer cases diagnosed between 2004 and 2016. We used the χ^2^ (Chi-square) test to compare differences between different categorical variables according to the number of TDs. The Cox proportional hazards regression model was used to determine the independent association of different clinical and pathological variables with CSS, which were adjusted for other significant prognostic factors.

**Results:**

We have identified 29,017 patients diagnosed with stage III colon cancer from the SEER database. The results of multivariate analyses showed that patients with the receipt of chemotherapy had 54.7% decreased risk of cancer-specific mortality compared with those not (HR = 0.453, 95% CI = 0.425–0.483, *P* < 0.0001) in the no-TD group; In the 1–2-TD group, patients with the receipt of chemotherapy had 56.8% decreased risk of cancer-specific mortality compared with those not (HR = 0.432, 95% CI = 0.364–0.512, *P* < 0.0001); In the ≥3-TD group, patients with the receipt of chemotherapy had 51.8% decreased risk of cancer-specific mortality compared with those not (HR = 0.482, 95% CI = 0.389–0.597, *P* < 0.0001).

**Conclusions:**

Our study demonstrated that the presence of TDs was associated with a dismal prognosis and high number of TDs would also contribute to the worse survival of colon cancer. High number of TDs did not affect the survival benefit of chemotherapy in stage III colon cancer.

## Introduction

Colon cancer is one of the most malignant tumors and occupies the fifth leading cause of cancer deaths worldwide (1). It is reported that more than one-third of colon cancer patients would present with lymph node metastases, that is, stage III colon cancer. Stage III colon cancer is considered to be an aggressive disease and has a clinically significant risk of distant metastasis after resection (2). 5-Fluorouracil (5-FU)-based chemotherapy regimens are commonly used in stage III colon cancer followed by surgical resection of the primary tumor, and it is known that 50% of the stage III colon cancer patients are cured by surgery alone, 20% with addition of adjuvant chemotherapy; however, 30% of those patients would experience recurrence, which is generally fatal within 2–3 years (3–6). Therefore, it is necessary to predict the efficacy of stage III colon cancer chemotherapy.

In 1935, tumor deposit (TD) was firstly reported in some node-negative colorectal cancer patients after meticulous pathological dissection of colorectal cancer specimens, and the researchers believed that these non-lymphatic metastases were the result of vascular spread (7). Over the years, the understanding of TDs was constantly changing, and the definition of TDs (also known as N1c) in colorectal cancer had been revised in the 8^*th*^ edition of the American Joint Committee on Cancer (AJCC) staging system. Tumor deposit was a discrete nodule of cancer in pericolic/perirectal fat or adjacent mesentery, without histological evidence of residual lymph node or identifiable vascular or neural structures (7, 8).

Tumor deposits had been considered as an indicator of poor prognosis in colorectal cancer (9–11). What is more, it was reported that patients with both TDs and lymph node metastasis would have a worse prognosis than patients with either alone (12, 13). However, no previous studies had evaluated the role of TDs in predicting the efficacy of chemotherapy in stage III colon cancer.

## Materials and Methods

### Data Source and Study Population

**FIGURE 1:**
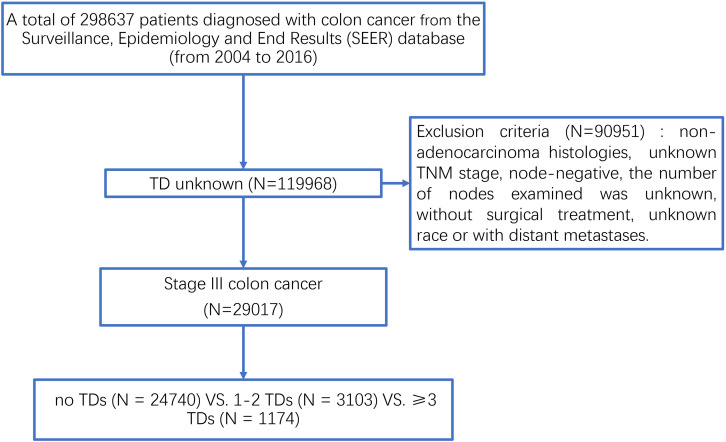
The selection process of patients diagnosed with stage III colon cancer from the SEER database.

Sponsored by the National Cancer Institute (NCI), the Surveillance, Epidemiology and End Results (SEER) database is representative of the United States population and consists of population-based cancer registries that cover approximately 28% of the United States population with patient-level data abstracted from 18 geographically diverse populations that represent rural, urban, and regional populations and is freely available for cancer-based epidemiology investigation and survival analysis (14). Using the SEER^∗^Stat software Version 8.3.6, we started with a national cohort of 298637 colon cancer cases diagnosed between 2004 and 2016 (Figure 1). Of these, we excluded patients without the exact number of TDs because we want to evaluate the clinical role of TDs in colon cancer. In addition, we also excluded some colon cancer patients with the following exclusion criteria: (1) unknown TNM stage, (2) non-adenocarcinoma histological type, (3) node-negative, (4) number of nodes examined was unknown, (5) without surgical treatment, and (6) unknown race and with distant metastases. Finally, only qualified patients diagnosed with stage III colon cancer were included in this study, and all the patients were divided into three groups: no TDs (*N* = 24740) vs. 1–2 TDs (*N* = 3103) vs. ≥3 TDs (*N* = 1174). We then ascertained variables of interest from the SEER database, including T stage (T1 stage, T2 stage, T3 stage, or T4 stage), N stage (N1 stage or N2 stage), age at diagnosis (years), race (white race, black race, or other race), gender (male or female), tumor grade (well/moderate tumor grade, poor/anaplastic tumor grade or unknown tumor grade), histological type (adenocarcinoma or mucinous/signet-ring cell carcinoma), the receipt of chemotherapy (no/unknown or yes), and the number of TDs (no TDs, 1–2 TDs, or ≥3 TDs).

### Statistical Analysis

We used the χ^2^ (Chi-square) test to compare differences between different categorical variables according to the number of TDs. Cancer-specific survival (CSS) was used as the outcome of interest, which was calculated from the date of diagnosis to the date of colon cancer death. The Cox proportional hazards regression model was used to determine the independent association of different clinical and pathological variables with CSS, which were adjusted for other significant prognostic factors. Moreover, only variables considered significant (*P* < 0.20) in univariable analyses would be incorporated into multivariate Cox models. An accumulated risk curve was also constructed by the Kaplan–Meier method to compared CSS of colon cancer patients according to the number of TDs. Significant differences in survival were tested with the log-rank tests. The 95% confidence intervals (CI) for hazard ratios (HRs) were generated and reported. All calculations were performed with SPSS 22.0 (Chicago, IL, United States), and all *P* values were two-sided and would be considered of statistical significance when *P* values were less than 0.05.

## Results

### Baseline Patient Characteristics

We have identified 29,017 patients diagnosed with stage III colon cancer with the known number of TDs from the SEER database, and all the patients were divided into three groups, including no TDs (*N* = 24740), 1–2 TDs (*N* = 3103), and ≥3 TDs (*N* = 1174). There were 12,581 (43.4%) patients with ≤65 years and 16,436 (56.6) with >65 years. The mean age of the study population was 67.41 years, and the median age was 68 years. Among the study population, 14,132 (48.7%) patients were males and 14,874 (51.3%) patients were females; 1412 (4.9%) patients were T1 stage, 2679 (4.9%) patients were T2 stage, 18,725 (9.2%) patients were T3 stage, and 6201 (64.5%) patients were T4 (21.4) stage; and 20,120 (69.3%) patients were N1 stage, 8897 (30.7%) patients were N2 stage.

The detailed clinicopathological characteristics of patients based on the number of TDs were summarized in Table 1. It was found that a higher number of TDs preferred to be associated with higher T stage (*P* < 0.001), higher N stage (*P* < 0.001), higher tumor grade (*P* < 0.001), and mucinous/signet-ring cell carcinoma (*P* = 0.001), showing that the presence of TDs preferred to be associated with aggressive features of pathology. However, age at diagnosis (*P* = 0.054), race (*P* = 0.249), gender (*P* = 0.197), and the receipt of chemotherapy (*P* = 0.106) between different TD groups did not achieve statistical significance.

**TABLE 1:** Demographic and clinicopathological characteristics of stage III colon cancer patients.

**Groups**	**Number of Patients (%)**			***P***
	**No TDs**	**1–2 TDs**	**≥3 TDs**	
T stage				< 0.001
T1	1340 (5.4)	63 (2.0)	9 (0.8)	
T2	2465 (10.0)	181 (5.8)	33 (2.8)	
T3	16054 (64.9)	1998 (64.4)	673 (57.3)	
T4	4881 (19.7)	861 (27.7)	459 (39.1)	
N stage				< 0.001
N1	17268 (69.8)	2234 (72.0)	618 (52.6)	
N2	7472 (30.2)	869 (28.0)	556 (47.4)	
Age at diagnosis (years)				0.054
≤65	10747 (43.4)	1297 (41.8)	537 (45.7)	
>65	13993 (56.6)	1806 (58.2)	637 (54.3)	
Race				
White	19139 (77.4)	2398 (77.3)	912 (77.7)	0.249
Black	3218 (13.0)	410 (13.2)	132 (11.2)	
Other	2383 (9.6)	295 (9.5)	130 (11.1)	
Gender				0.197
Male	12008 (48.5)	1539 (49.6)	596 (50.8)	
Female	12723 (51.5)	1564 (50.4)	578 (49.2)	
Grade				< 0.001
Well/moderate	18039 (72.9)	2299 (74.1)	753 (64.1)	
Poor/anaplastic	6292 (25.4)	757 (24.4)	408 (34.8)	
Unknown	409 (1.7)	47 (1.5)	13 (1.1)	
Histology				0.001
Adenocarcinoma	22162 (89.6)	2818 (90.8)	1020 (86.9)	
Mucinous/signet-ring cell carcinoma	2578 (10.4)	285 (9.2)	154 (13.1)	
Chemotherapy				0.106
No/unknown	9682 (39.1)	1241 (40.0)	428 (36.5)	
Yes	15058 (60.9)	1862 (60.0)	746 (63.5)	

### The Prognosis of TDs in Stage III Colon Cancer

**FIGURE 2:**
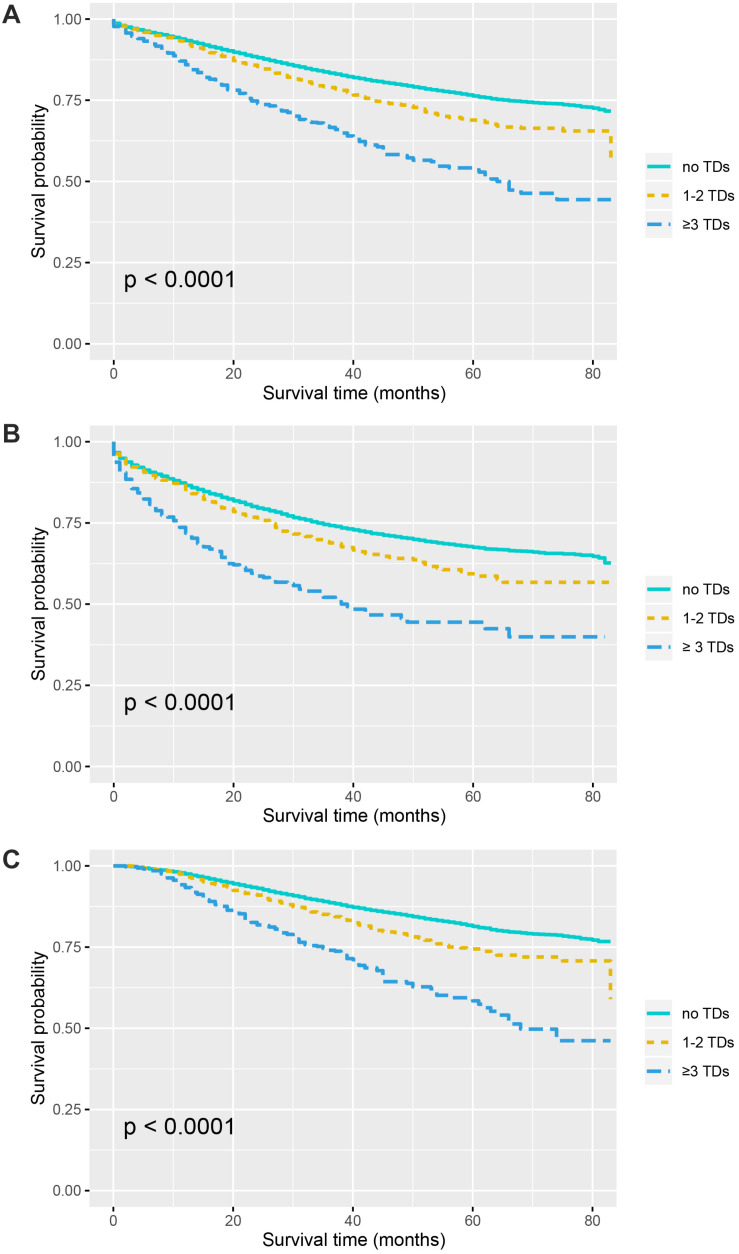
Survival curves for patients stratified by the number of TDs in panel **(A)** all the patients. **(B)** Patients without chemotherapy. **(C)** Patients treated with chemotherapy.

Kaplan–Meier curves of CSS according to the number of TDs are shown in Figure 2. It was found that the presence of TDs would reduce CSS of colon cancer patients: in all the stage III colon cancer patients, the 5-year CSS rates of no TDs, 1–2 TDs, and ≥3 TDs were 76.3, 68.9, and 53.6%, respectively (*P* < 0.0001, Figure 2A); in the stage III colon cancer patients without the receipt of chemotherapy, the 5-year CSS rates of no TDs, 1–2 TDs, and ≥3 TDs were 67.5, 59.4, and 44.4%, respectively (*P* < 0.0001, Figure 2B); in the stage III colon cancer patients with the receipt of chemotherapy, the 5-year CSS rates of no TDs, 1–2 TDs, and ≥3 TDs were 81.4, 74.4, and 58.4%, respectively (*P* < 0.0001, Figure 2C).

### The Role of Tumor Deposits in Predicting the Efficacy of Chemotherapy in Stage III Colon Cancer

**FIGURE 3:**
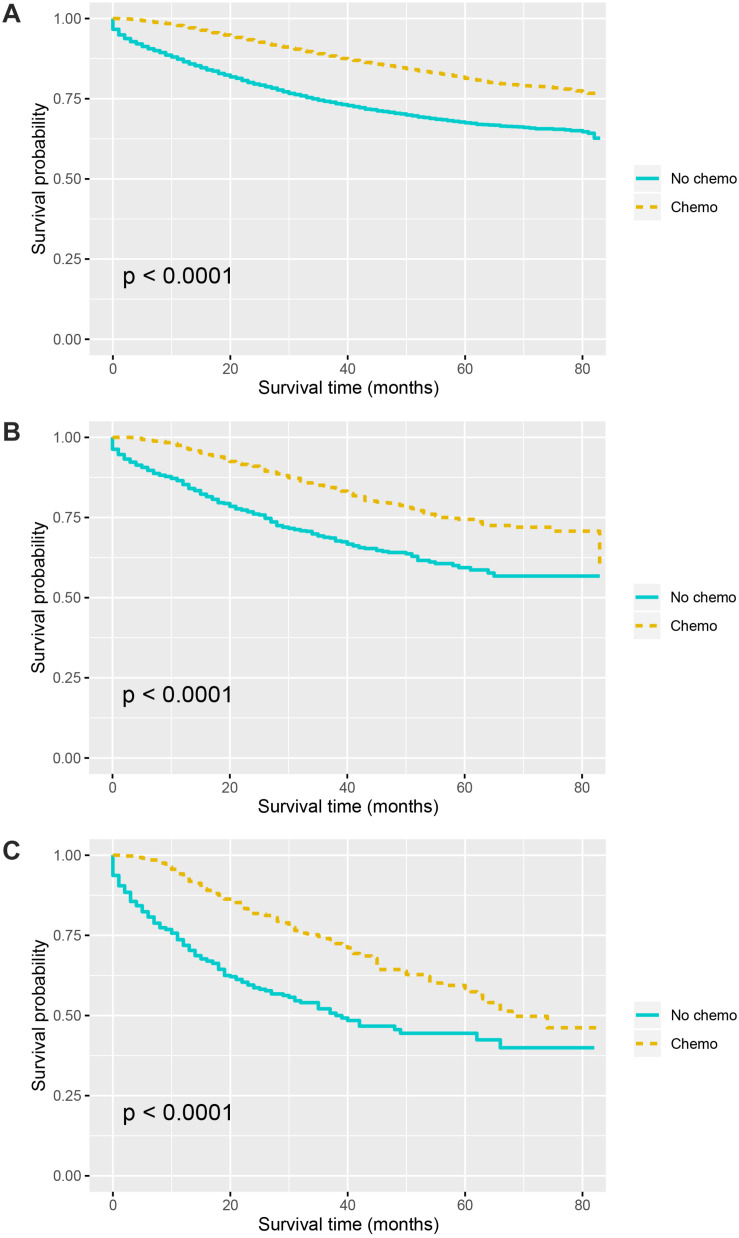
Survival curves for patients stratified by the receipt of chemotherapy or not in colon cancer patients with **(A)** No TDs. **(B)** 1–2 TDs. **(C)** ≥3 TDs.

Kaplan–Meier curves of CSS according to the number of TDs are shown in Figure 3. It was found that the receipt of chemotherapy would significantly improve CSS of stage III colon cancer patients: in the no-TD group, the 5-year CSS rates of patients without and with the receipt of chemotherapy were 67.5 and 81.4%, respectively (*P* < 0.0001, Figure 3A); in the 1–2-TD group, the 5-year CSS rates of patients without and with the receipt of chemotherapy were 59.4 and 74.4%, respectively (*P* < 0.0001, Figure 3B); in the ≥3-TD group, the 5-year CSS rates of patients without and with the receipt of chemotherapy were 44.4 and 58.4%, respectively (*P* < 0.0001, Figure 3C). In addition, the Cox proportional hazards regression model was also used to verify the above findings. In the no-TD group, the univariate Cox analysis produced eight variables, which were then incorporated into multivariate Cox models, including T stage, N stage, age at diagnosis, race, gender, tumor grade, histological type, and the receipt of chemotherapy. What is more, the results of multivariate analyses showed that patients with the receipt of chemotherapy had 54.7% decreased risk of cancer-specific mortality compared with those not (HR = 0.453, 95% CI = 0.425–0.483, *P* < 0.0001; Table 2) in the no-TD group. In the 1–2-TD group, univariate Cox analysis produced seven variables, which were then incorporated into multivariate Cox models, including T stage, N stage, age at diagnosis, race, gender, tumor grade, and receipt of chemotherapy. What is more, the results of multivariate analyses showed that patients with receipt of chemotherapy had 56.8% decreased risk of cancer-specific mortality compared with those not (HR = 0.432, 95% CI = 0.364–0.512, *P* < 0.0001; Table 3) in the 1–2-TD group. In the ≥3-TD group, the univariate Cox analysis produced six variables, which were then incorporated into multivariate Cox models, including T stage, N stage, age at diagnosis, tumor grade, histology and the receipt of chemotherapy. What is more, the results of multivariate analyses showed that patients with the receipt of chemotherapy had 51.8% decreased risk of cancer-specific mortality compared with those not (HR = 0.482, 95% CI = 0.389–0.597, *P* < 0.0001; Table 4) in the ≥3-TD group.

**TABLE 2:** HRs of different demographic and clinicopathological characteristics in stage III colon cancer with no tumor deposits.

**Groups**	**Univariate analyses**		**Multivariate analyses**	
	**HR (95%CI)**	***P***	**HR (95%CI)**	***P***
T stage		< 0.001		< 0.001
T1			1	
T2			1.654 (1.222–2.238)	0.001
T3			3.380 (2.585–2.238)	< 0.001
T4			7.306 (5.572–9.579)	< 0.001
N stage		< 0.001		< 0.001
N1			1	
N2			1.807 (1.698–1.924)	
Age at diagnosis (years)		< 0.001		< 0.001
≤65			1	
>65			1.439 (1.345–1.540)	
Race		0.027		< 0.001
White			1	
Black			1.257 (1.150–1.374)	< 0.001
Other			0.933 (0.839–1.037)	0.199
Gender		0.018		0.974
Male			1	
Female			0.999 (0.940–1.062)	
Grade		< 0.001		< 0.001
Well/Moderate			1	
Poor/Anaplastic			1.369 (1.282–1.461)	< 0.001
Unknown			1.328 (1.058–1.665)	0.014
Histology		< 0.001		0.055
Adenocarcinoma			1	
Mucinous/signet-ring cell carcinoma			1.093 (0.998–1.198)	
Chemotherapy		< 0.001		< 0.001
No/unknown			1	
Yes			0.453 (0.425–0.483)	

**TABLE 3:** HRs of different demographic and clinicopathological characteristics in stage III colon cancer with 1–2 tumor deposits.

**Groups**	**Univariate analyses**		**Multivariate analyses**	
	**HR (95%CI)**	***P***	**HR (95%CI)**	***P***
T stage		< 0.001		< 0.001
T1			1	
T2			0.863 (0.364–2.045)	0.738
T3			1.361 (0.642–2.883)	0.421
T4			2.859 (1.343–6.083)	0.006
N stage		< 0.001		< 0.001
N1			1	
N2			1.798 (1.520–2.127)	
Age at diagnosis (years)		< 0.001		< 0.001
≤65			1	
>65			1.517 (1.266–1.819)	
Race		0.105		0.002
White			1	
Black			1.468 (1.171–1.841)	0.001
Other			0.911 (0.680–1.221)	0.534
Gender		0.079		0.435
Male			1	
Female			1.066 (0.907–1.253)	
Grade		< 0.001		< 0.001
Well/moderate			1	
Poor/anaplastic			1.429 (1.200–1.701)	< 0.001
Unknown			0.462 (0.191–1.118)	0.087
Histology		0.589		
Adenocarcinoma				
Mucinous/signet-ring cell carcinoma				
Chemotherapy		< 0.001		< 0.001
No/unknown			1	
Yes			0.432 (0.364–0.512)	

**TABLE 4:** HRs of different demographic and clinicopathological characteristics in stage III colon cancer with ≥3 tumor deposits.

**Groups**	**Univariate analyses**		**Multivariate analyses**	
	**HR (95%CI)**	***P***	**HR (95%CI)**	***P***
T stage		< 0.001		< 0.001
T1			1	
T2			0.367 (0.023–5.903)	< 0.001
T3			2.801 (0.390–20.123)	0.479
T4			4.341 (0.603–31.238)	0.306
N stage		< 0.001		0.145
N1			1	
N2			1.848 (1.484–2.301)	
Age at diagnosis (years)		< 0.001		< 0.001
≤65			1	
>65			1.662 (1.332–2.074)	
Race		0.453		
White				
Black				
Other				
Gender		0.325		
Male				
Female				
Grade		< 0.001		0.002
Well/moderate			1	
Poor/anaplastic			1.474 (1.179–1.843)	0.001
Unknown			1.981 (0.906–4.331)	0.087
Histology		0.018		0.511
Adenocarcinoma			1	
Mucinous/signet-ring cell carcinoma			1.105 (0.820–1.488)	
Chemotherapy		< 0.001		< 0.001
No/Unknown			1	
Yes			0.482 (0.389–0.597)	

## Discussion

In 1935, TD was firstly reported in some node-negative colorectal cancer patients after meticulous pathological dissection of colorectal cancer specimens, and in 1997, TD was first introduced into the AJCC TNM staging system (7). In our study, a total of 4277 (14.7%) stage III colon cancer patients were diagnosed with TDs, and the proportion was a little lower than in the previous study (15). It was reported by some researches that, some variables, such as T4 and N2, which were definitively associated with worse survival expectations, were found to occur more frequently in stage III colon cancer with TDs (9–11). In our analyses, it was found that a higher number of TDs preferred to be associated with higher T stage, higher N stage, higher tumor grade, and mucinous/signet-ring cell carcinoma, indicating that the presence of TDs preferred to be associated with aggressive features of pathology, which we believed will provide further understanding of the nature and origin of TDs.

The most novel finding of a study from Canada had shown that a high number of TDs (≥3) were indicative of worse prognosis than 1 to 2 TDs; therefore, all patients diagnosed with TDs in the present study were divided into two subgroups, including the 1–2-TD and ≥3-TD groups. In addition, the present study also investigated the prognostic value of TDs in different TD subgroups. Recently, a retrospective analysis had found that the survival difference between N1b and N1c did not achieve statistical difference; what is more, N1c was associated with worse prognosis compared to N1a (16). Moreover, the results of survival analyses in this study showed that the presence of TDs would reduce CSS of colon cancer patients: in all the stage III colon cancer patients, the 5-year CSS rates of no TDs, 1–2 TDs, and ≥3 TDs were 76.3, 68.9, and 53.6%, respectively (*P* < 0.0001); in the stage III colon cancer patients without the receipt of chemotherapy, the 5-year CSS rates of no TDs, 1–2 TDs, and ≥3 TDs were 67.5, 59.4, and 44.4%, respectively (*P* < 0.0001); in the stage III colon cancer patients with the receipt of chemotherapy, the 5-year CSS rates of no TDs, 1–2 TDs, and ≥3 TDs were 81.4, 74.4, and 58.4%, respectively (*P* < 0.0001). The subgroup analyses in our study indicated that the presence of TDs was associated with a dismal prognosis and a high number of TDs would also contribute to worse survival, which was in agreement with previous findings that some previous studies had demonstrated that TD was an adverse prognostic factor in colon cancer (15, 17, 18). Moreover, in 2018, it was reported by a recent study that the presence of TDs had 220% increased risk of developing disease recurrence (2).

It should also be noted that the origins of TDs were diverse. By serial sectioning in a series of 30 irregular TDs from 418 stage III colon adenocarcinomas patients, it was found that almost 40% of all the TDs showed a combined perineural, perivascular, and intravascular origin. In addition, a perineural origin was present in 77% of cases and an intravascular origin in 83% of cases (19). In 2010, Wünsch and his colleagues conducted a study with 69 TDs and it also showed the similar diversity to the above finding (20). The worse prognosis of patients diagnosed with TDs was partly explained by the presence of vessels and nerves in TDs, because it would add more anatomic highways for the metastases and spread of tumor cells (21).

In the Kaplan–Meier CSS analyses according to the number of TDs in the current study, it was found that the receipt of chemotherapy would significantly improve CSS of stage III colon cancer patients: in the no-TD group, the 5-year CSS rates of patients without and with the receipt of chemotherapy were 67.5 and 81.4%, respectively (*P* < 0.0001); in the 1–2-TD group, the 5-year CSS rates of patients without and with the receipt of chemotherapy were 59.4 and 74.4%, respectively (*P* < 0.0001); in the ≥3 TD group, the 5-year CSS rates of patients without and with the receipt of chemotherapy were 44.4 and 58.4%, respectively (*P* < 0.0001). The efficacy of adjuvant chemotherapy in stage III colon cancer had been widely recognized in previous studies, and it was also confirmed in different TD subgroups of our study (22, 23).

What is more, the main significance of this study was to evaluate the survival benefit difference in stage III colon cancer with high number of TDs. Moreover, we have found that, in the no TD group, the Cox proportional hazards regression analyses showed that patients with the receipt of chemotherapy had 54.7% decreased risk of cancer-specific mortality compared with those not. In the 1–2-TD group, patients with the receipt of chemotherapy had 56.8% decreased risk of cancer-specific mortality compared with those not. In the ≥3-TD group, similarly, the results of multivariate analyses showed that patients with the receipt of chemotherapy had 51.8% decreased risk of cancer-specific mortality compared with those not.

Tumor deposits have become a hotspot in colon cancer study during recent years, and it was demonstrated that a high number of TDs was indicative of worse prognosis (21, 24). In 2017, Nagtegaal et al. (21) proposed that TDs and their actual number were equal to the number of lymph node metastases in making treatment decisions and the number of TDs should be fully included in the TNM staging. To our knowledge, however, few studies investigated the efficacy of survival benefit in stage III colon cancer with high number of TDs. In the present study, we demonstrated that, for the first time, the efficacy of chemotherapy was similar in different TD subgroups and a high number of TDs did not affect the survival benefit of chemotherapy in stage III colon cancer.

We also need to address the shortcomings in the current study. The first was the inherent weakness SEER data set, such as the lack of some detailed clinicopathological characteristics, which might introduce some selection biases in our study. The other limitation in our work was that our study was a retrospective one. In addition, patients without the information of TDs of some patients are excluded from our analyses, which might cause the selection bias. More researches, especially large prospective ones, were needed in the future to generalize the results.

In summary, it was found that the presence of TDs was associated with a dismal prognosis and a high number of TDs would also contribute to the worse survival of colon cancer. Moreover, we demonstrated that, for the first time, the efficacy of chemotherapy was similar in different TD subgroups and high number of TDs did not affect the survival benefit of chemotherapy in stage III colon cancer.

## Data Availability Statement

Publicly available datasets were analyzed in this study. This data can be found here: https://seer.cancer.gov/.

## Author Contributions

YR was responsible for the conception and design of this study. MS, JW, CZ, and XZ performed the study selection, data extraction, and statistical analyses. MS, HZ, and GY performed the literature search and wrote the first draft of the manuscript. MS, HZ, and YR revised and edited the final version of manuscript. All authors reviewed and approved the submitted version of the manuscript.

## Conflict of Interest

The authors declare that the research was conducted in the absence of any commercial or financial relationships that could be construed as a potential conflict of interest.
